# Human Herpesvirus 8 and Pulmonary Hypertension

**DOI:** 10.3201/eid1109.040880

**Published:** 2005-09

**Authors:** Emanuele Nicastri, Carmine Dario Vizza, Fabrizio Carletti, Stefania Cicalini, Roberto Badagliacca, Roberto Poscia, Giuseppe Ippolito, Francesco Fedele, Nicola Petrosillo

**Affiliations:** * National Institute for Infectious Diseases IRCCS Lazzaro Spallanzani, Rome, Italy;; † "La Sapienza" University, Rome, Italy

**Keywords:** HHV-8, pulmonary hypertension, dispatch

## Abstract

Human herpesvirus 8 (HHV-8) antibodies were detected in 1 of 33 patients with pulmonary hypertension (including in 1 of 16 with idiopathic pulmonary arterial hypertension), 5 of 29 with cystic fibrosis, and 3 of 13 with interstitial lung disease. No relationship between HHV-8 infection and pulmonary hypertension was found.

Human herpesvirus 8 (HHV-8) has been detected in patients with Kaposi sarcoma, primary-effusion B-cell lymphomas, and Castleman disease (*1*). Recently, 2 articles from 1 group suggested that HHV-8 has a role in the pathogenesis of idiopathic pulmonary arterial hypertension (IPAH) (*2**,**3*). IPAH has been reported in 2 patients with HHV-8–associated Castleman disease; lung tissue from 1 of these patients was positive for latency-associated nuclear antigen-1 (*3*). HHV-8 latency-associated nuclear antigen-1 and HHV-8 viral cyclin gene were identified in the lung tissue of 10 (62.5%) of 16 patients with IPAH, whereas only 1 (7.1%) of 14 patients with associated pulmonary hypertension (PH) had HHV-8 gene sequences in lung tissue (*2*). Conversely, Japanese researchers failed to confirm the detection of HHV-8 latency-associated nuclear antigen-1 in 10 IPAH patients (*4*).

Identifying HHV-8 as a cofactor in IPAH pathogenesis could raise relevant therapeutic and preventive issues. We conducted a seroprevalence study aimed at detecting antibodies to HHV-8 among lung transplantation candidates; we assessed the HHV-8 seroprevalence among PH patients with and without IPAH, and we compared results with those of non-PH patients.

## The Study

We retrospectively analyzed data from 75 patients referred to the Department of Cardiovascular and Respiratory Sciences of the University of Rome La Sapienza from January 2001 to February 2004 for clinical and serologic (hepatitis C virus, hepatitis B virus, HHV-8, and cytomegalovirus) evaluation for lung transplantation. The diagnosis of PH was based on international criteria (*5**,**6*). Echocardiographic data, including the right ventricular end-diastolic diameter (RVEDD), were available for all patients. Informed consent for medical and surgical procedures was obtained for all enrolled patients.

Thirty-three of 75 patients had significant PH (mean pulmonary arterial pressure [PAP] >25 mm Hg assessed by right heart catheterization). Sixteen of them had IPAH (PAP 53.4 ± 17.1 mm Hg), whereas among the 17 patients with secondary PH, 7 patients had chronic thromboembolic PH (PAP 40.3 ± 7.8), and the remaining 10 patients (PAP 37.1 ± 12.2 mm Hg) had PH associated with connective tissue disease (4 patients), HIV infection (3 patients), and lung disease (3 patients). The 42 patients without PH included 29 patients with cystic fibrosis (PAP 21.1 ± 3.3 mm Hg) and 13 patients with interstitial lung disease (PAP 18 ± 4.6 mm Hg) (8 patients with idiopathic pulmonary fibrosis, 2 with sarcoidosis, 3 with pulmonary fibrosis secondary to bleomycin treatment).

We performed assays for antibodies directed to lytic antigens of HHV-8 in plasma samples, according to a previously well-described method (*7*). Briefly, we used an in-house indirect immune fluorescent assay based on BCBL-1 cell line. Samples reactive at 1:40 dilution in the antilytic test were considered positive. As assessed in a large-scale multicenter study that employed a consensus-based method for defining the "true" status of specimens, this assay had the highest sensitivity of the assays evaluated (97.1%) and a specificity of 83.2% (*8*).

## Conclusions

The 75 patients (38 male), who were candidates for lung transplantation, were all born and living in Italy. Their mean age was 40.5 years (range 14–74). Antibodies against lytic antigens of HHV-8 were detected in 9 (12.0%) patients (median HHV-8 antibody titer 1:160, range 1:80–1:320).

No significant differences in age, sex, current residency, and cardiopulmonary symptoms (e.g., dyspnea, orthopnea, peripheral edema) were found between patients with or without HHV-8 antibodies. Nevertheless, patients with HHV-8 antibodies were generally younger (36.1 years ± 13.0 vs. 41.0 ± 14.3 years, p = 0.3) and more likely to be male (6 [66.7%] of 9 vs. 35 [50.7%] of 69, p = 0.4 ) than patients with no HHV-8 infection. All 3 patients with HIV infection were HHV-8 negative.

A higher heart rate and lower RVEDD, evaluated by echocardiography, were found in HHV-8–seropositive patients compared to HIV–seronegative patients (112 ± 20 vs. 89 ± 15 beats/min, p<0.001 and 22.8 ± 4.5 mm vs. 30.9 ± 6.9 mm, p = 0.02, by analysis of variance). No further difference in echocardiographic parameters was reported between patients with or without HHV-8 antibodies.

A difference in the HHV-8 seroprevalence was found between the PH patients (3.0%) and the patients without PH (19.0%). Patients with PH were older (47.3 years ± 12.3 vs. 33.9 ± 12.7 years, p<0.001) and more like to be male (9 [27.3%] of 33 and 30 [71.4%] of 42, p<0.001). Among the 33 patients with PH, 1 (6.3%) of 16 with IPAH had serologic HHV-8 antibodies, whereas no patient with secondary PH had HHV-8 antibodies (Table).

**Table:** Human herpesvirus 8 (HHV-8) seroprevalence among candidate patients for lung transplantation

Diagnosis	Patients	Female sex (%)	Median age (range)	HHV-8 seroprevalence (%)
Patients with pulmonary hypertension				
Idiopathic pulmonary arterial hypertension	16	14 (87.5)	46 (28–74)	1 (6.3)
Secondary pulmonary hypertension	17	11 (64.7)	44 (22–65)	0 (0)
Patients without pulmonary hypertension				
Cystic fibrosis	29	9 (31.0)	23 (14–28)	5 (17.2)
Interstitial lung disease	13	3 (23.1)	47 (43–74)	3 (23.1)

Among the 42 patients with no clinical or diagnostic evidence of PH, 5 (17.2%) of the 29 with cystic fibrosis and 3 (23.1%) of the 13 with interstitial lung disease had HHV-8 antibodies, all of them affected by idiopathic pulmonary fibrosis. No difference in HHV-8 seroprevalence rate was found in patients with cystic fibrosis and in patients with interstitial lung disease (Table).

We found an 11.5% prevalence of HHV-8 antibodies and a 1:160 median HHV-8 antibody titer among a population of Italian patients who were candidates for lung transplantation. The seroprevalence and the range of the median end point dilution are similar to those found in the Italian general population of blood donors, <1:40–1:160 (*8**,**9*).

Our findings are different from those found by 1 group of researchers (*2**,**3*) among IPAH patients and consistent with other results (*4**,**10*). HHV-8 antibodies were detected more frequently among young boys and patients without PH. IPAH patients had a low HHV-8 seroprevalence rate (6.3%) with a female prevalence (87.5%). The HHV-8 prevalence rate in Europe and the United States is higher in men who have sex with men than in the general population (*11*), whereas consistent with our results, the specific literature on PH shows a female predominance in IPAH patients (1.7 female/male ratio) (*6*).

The higher heart rate observed in patients with HHV-8 antibodies is likely due to the higher prevalence of patients with cystic fibrosis in this subgroup (69%). Conversely, the higher RVEDD observed in patients without HHV-8 antibodies is likely related to the higher prevalence of diseases, such as IPAH and chronic thromboembolic PH, that cause severe right ventricular dysfunction in these patients. The limited sample size of the population does not allow an appropriate relevant analysis, but similar findings were found in a previous study (*12*) and could be related to an hyperdynamic circulation due to chronic infection.

Several limitations of our study should be mentioned. First, the high prevalence of HHV-8 antibodies among non-PH lung transplantation candidates could be the result of these patients' pulmonary disease and of their previous exposures to medical and surgical procedures not investigated in this study. Indeed, as previously reported (*13*), HHV-8 DNA was detected with significantly higher frequency in lung tissue samples of patients affected by idiopathic pulmonary fibrosis. Second, we separated IPAH from PH in the setting of autoimmune disease. We recognize that distinguishing IPAH from secondary PH is not always possible because underlying autoimmune disease can go undiagnosed. Additionally, the pulmonary and systemic pathologic features of the lung diseases being compared differ, even if the result is IPAH. This observation may affect some of the clinical physiologic parameters reported. In conclusion, demographic and virologic issues did not provide evidence of a direct relationship between HHV-8 infection and PH, either idiopathic or secondary.
